# Genome Halving with an Outgroup

**Published:** 2007-02-21

**Authors:** Chunfang Zheng, Qian Zhu, David Sankoff

**Affiliations:** 1 Department of Biology, University of Ottawa, Ottawa, Ontario, Canada; 2 Department of Biochemistry, University of Ottawa, Ottawa, Ontario, Canada; 3 Department of Mathematics and Statistics, University of Ottawa, Ottawa, Ontario, Canada

**Keywords:** tetraploid, genome doubling, cereals, genome rearrangement, synteny, algorithms

## Abstract

Some genomes are known to have incurred a genome doubling (tetraploidization) event in their evolutionary history, and this is reflected today in patterns of duplicated segments scattered throughout their chromosomes. These duplications may be used as data to “halve” the genome, i.e. to reconstruct the an cestral genome at the moment of tetraploidization, but the solution is often highly non-unique. To resolve this problem, we adapt the genome halving algorithm of El-Mabrouk and Sankoff to take account of an external reference genome. We apply this to reconstruct the tetraploid ancestor of maize, using either rice or sorghum as the reference.

## Introduction

Many genomes have been shown to result from an ancestral doubling, or tetraploidization, event, followed by a period of diploidization, i.e. the loss of compartmentalization between the two original copies of the genome, as well as genome rearrangement through intra-and interchromosomal movement of genetic material. The genome halving problem is to reconstruct the ancestral genome on the basis of a decomposition of the present-day genome into a set of apparently duplicated blocks of genes or DNA sequence dispersed among the chromosomes. A quantitative approach to this problem was first discussed by Seoighe and Wolfe (1998) in the context of the genome doubling of the ancestor of the yeast *Saccharomyces cerevisiae*. At the same time, motivated by studies of genome duplication in early vertebrates (Nadeau and Sankoff, 1997), El-Mabrouk and colleagues (1998, El-Mabrouk and colleagues 1999a, El-Mabrouk and colleagues 1999b) published a series of papers on the combinatorial optimization approach to the problem, culminating in a general solution (El-Mabrouk and Sankoff, 2003). Further refinements have been published by Alekseyev and Pevzner (2004).

Seoighe and Wolfe (1998) noted the extreme non-uniqueness associated with the solution to the genome halving problem and suggested that this difficulty could be attenuated through the use of a reference genome, or outgroup. The suggestion to use a reference genome was taken up to study the post-tetraploidization evolution of *S. cerevisiae*, both in reference to the genome of *Ashbya gossypii* (Dietrich et al. 2004) and to that of *Kluyveromyces waltii* (Kellis et al. 2004), though without recourse to genome rearrangement or genome halving algorithms. Similar research compared mammalian genomes with the tetraploid ancestor of the pufferfish *Tetraodon nigroviridis* (Jaillon et al. 2004). In the present paper, we formalize this strategy by developing a general algorithm to reconstruct an ancestral tetraploid genome with reference to an outgroup genome. We apply it to infer the ancestor of the maize (*Zea mays*) genome, with the rice (*Oryza sativa*) and sorghum (*Sorghum bicolor*) genomes as outgroups. For this purpose, we are concerned only with duplicated blocks in maize, and their single-copy counterparts in rice and sorghum, as extracted from the Gramene database (Jaiswal et al. 2006), and not the rest of the genomes.

Our strategy is to generate all the solutions to the genome halving problem for the maize genome, and to focus on the subset of these that have a minimum rearrangement distance with the rice (or sorghum) genome. We formulate a search heuristic to transcend the set of optimal halving solutions to find the most realistic ancestral genome that minimizes the sum of the distance between the ancestral tetraploid and present-day maize and the distance between rice (or sorghum) and the diploid form of the ancestor.

## The Data

It is generally agreed that the maize genome underwent a genome doubling event some 11–16 million years ago (Gaut and Doebley, 1997). While some duplicated regions clearly attest to this event, there is no consensus on the exact inventory of such regions. Moore et al. (1995) and Wilson et al. (1999) presented two largely consistent views of syntenic blocks across the cereals based on the mapping evidence at the time. These included 14 and 19 duplicated blocks in the maize genome. Gaut (2001) gave a more comprehensive account of the pattern of 23 duplicated regions, based on maize genomic sequence data in 2001. He did not completely establish the relative position of all the syntenies on the chromosomes in this work.

Even now that the rice genome has been sequenced, and the maize genome project is well-advanced, it is no trivial matter to identify the duplicate blocks resulting from the tetraploidization event. The maize genome has many other duplicated segments dating from periods both after and before the tetraploidization and even before the divergence from the other cereals. This is complicated by post-tetraploidization genome rearrangement events, deletions and insertions of genetic material, transpositions of genes or larger segments from one site on the genome to another, and loss of homology between the parts of the duplicated regions.

**Figure 1:**
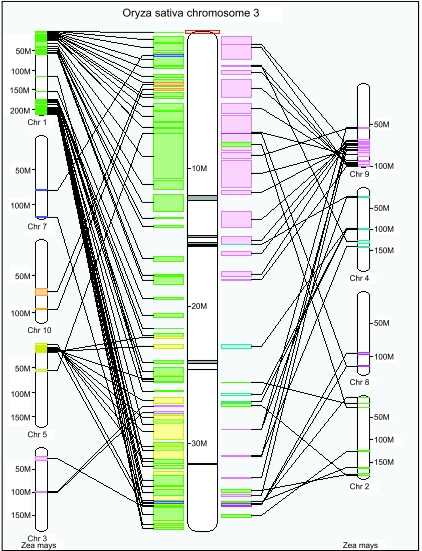
Syntenies between rice chromosome 1 and maize chromosomes, as produced by Gramene.

The databank which has the most information on the syntenies among the cereal genomes is Gramene (Jaiswal et al. 2006). The current version at time of writing is release 21. From this we can obtain a conservative (i.e. confined to high homology regions only) estimate of duplicate blocks by comparison with the rice genome. For example, in Figure 1, we can visually identify large duplicated regions in chromosomes 1 and 9, chromosomes 1 and 5, and possibly a number of smaller ones, all by virtue of their common homology with regions of rice chromosome 3.

**Figure 2:**
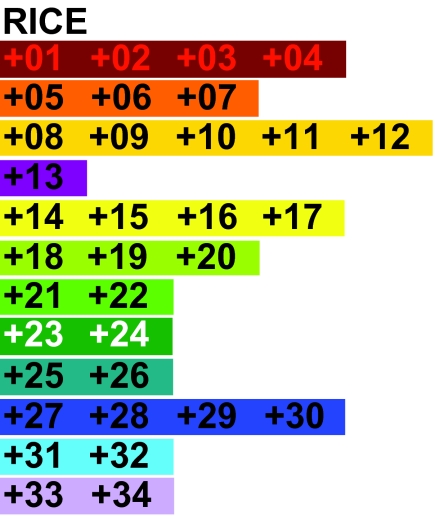

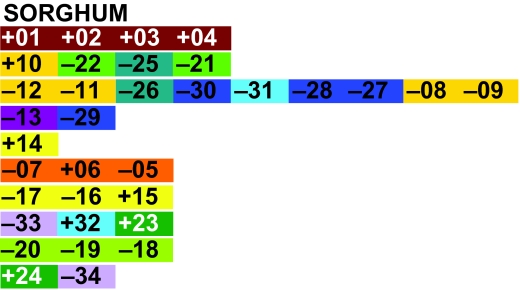

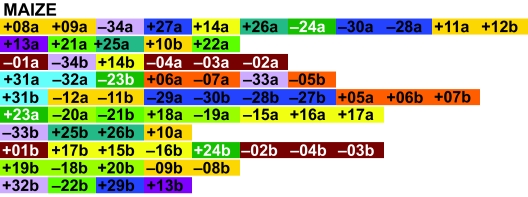
Order of syntenic blocks in rice, sorghum and, in two copies each, maize.

Unfortunately, there is as yet no comparison of syntenic blocks between sorghum and the other genomes on Gramene. However, there are extensive mapping data of various kinds of markers. We bolstered our preceding data collection by searching sets of duplicate markers in maize that had single copies in sorghum and rice, comparing mainly the Patterson, 2003, genetic map for sorghum, the IBM2 Neighbours, 2004 and Cornell Wilson, 1999, genetic maps for maize and the Annotated Nipponbare Sequence, 2006, sequence map for rice. All the markers satisfying these criteria fell into the rice-maize syntenies established previously. Based on these criteria, i.e. markers identified as homologous in Gramene, with a single copy in each of rice and sorghum and two copies in maize, plus the requirement that the maize and rice copies fall into the appropriate, previous identified, rice-maize syntenic blocks, we could now identify 34 syntenic blocks as basic data for our reconstruction. These data are depicted in Figure 2, but should be considered to constitute a working hypothesis; definitive data must await the finishing of the maize genome, the sequencing of the sorghum genome, and the further application of global alignment and synteny block construction methods.

## The Genome Halving Algorithm

Distance based on genomic structure *d*(*X,Y*) is calculated by rapid, albeit complicated, rearrangement algorithms for finding the minimum number of operations necessary to convert one genome *X* into another *Y*. The genomes are represented by signed permutations on 1,···, *n* and the biologically-motivated operations generally include inversions (implying as well change of sign, i.e. change of strand) of chromosomal segments, reciprocal translocations (of telomere-containing segments of two chromosomes) and chromosome fission or fusion. They may also include transpositions (including “jumping genes”) of segments from one site to another on a chromosome or interchanges of segments on a chromosome, both of which count as two steps compared to one for the previously mentioned operations.

Rearrangement algorithms (e.g. Tesler, 2002) make use of the bi-coloured “breakpoint graph” or similar structure, where each end of an oriented syntenic block, gene or marker on genome *X* is joined by a red edge to the adjoining end of the adjacent syntenic block, gene or marker, and these same ends, represented by the 2*n* vertices in the graph are joined by black edges determined by the adjacencies in genome *Y*. The breakpoint graphs necessarily consist of disjoint alternating cycles and/or paths, and it can be shown that *d* (*X,Y*) = *n* − *c*, where *c* is the number of cycles (in the case *X* and *Y* consist of single circular chromosomes), or *d* (*X,Y*) = *n* + χ − *c* −Π, where χ is the maximum number of linear chromosomes in *X* and *Y*, Π and counts the number of certain kinds of paths in the graph. The actual operations, *d* (*X,Y*) in number, may be reconstructed by splitting large cycles in the breakpoint graph into two cycles each, until there are *d* (*X,Y*) cycles each made up of two vertices, one red edge and one black edge. Every time a cycle is split, this corresponds to one rearrangement operation.

In the rearrangement algorithms, construction of the breakpoint graph is an easy preliminary step. The genome halving algorithms (El-Mabrouk and Sankoff, 2003; Alekseyev and Pevzner, 2004) also make use of the breakpoint graph, but the problem here is building the breakpoint graph where one of the genomes (the tetraploid) is unknown. This is done by segregating the vertices of the graph in a natural way into subsets, such that the vertices of all cycles must fall within a single subset, and then constructing these cycles in an optimal way within each subset so that the red edges correspond to the structure of the known genome and the black edges define the adjacencies of a tetraploid.

## A Heuristic for Minimizing *d*(*U, A*) + *d*(A ⊕ *A,T*)

Let *T* be a genome consisting of χ chromosomes and 2*n* genes, syntenic blocks or other markers, *g*_1,1_,···, *g*_1,_*_n_*; *g*_2,1_,···, *g*_2,_*_n_*, dispersed in any order on the chromosomes. For each *i*, we call *g*_1,_*_i_* and *g*_2,_*_i_* “duplicates,” but there is no particular property distinguishing elements of the set of *g*_1,_*_i_* from the set of *g*_2,_*_i_*. A potential “ancestral tetraploid” of *T* is written *A* ⊕ *A*, and consists of 2Ψ chromosomes, where some half (Ψ) of the chromosomes contains exactly one of each of *g*_1,_*_i_* or *g*_2,_*_i_* for each *i* =1,···, *n*. The remaining Ψ chromosomes are each identical to one in the first half, in that where *g*_1,_*_i_* appears on a chromosome in the first half, *g*_2,_*_i_* appears on the corresponding chromosome in the second half, and where *g*_2,_*_i_* appears in the first half, *g*_1,_*_i_* appears in the second. We define *A* to be either of the two halves of *A* ⊕ *A*, where the subscript 1 or 2 is suppressed from each *g*_1,_*_i_* or *g*_2,_*_i_*. These Ψ chromosomes, and the *n* genes, syntenic blocks or markers they contain, *g*_1_, ···, *g_n_* constitute a potential “ancestral diploid” of *T*.

A solution of the genome halving problem for *T* is any *A* such that *d* (*A* ⊕ *A*, *T*) is minimal.

Any genome *U* is a reference genome for *T* if it contains the *n* genes, syntenic blocks or markers *g*_1_,···, *g_n_*.

Let *U* be a reference genome for *T*. The central problem in this paper is to find a potential ancestral diploid genome *A* such that *d* (*U, A*) + *d* (*A* ⊕ *A,T*) is minimized.

**Figure 3:**
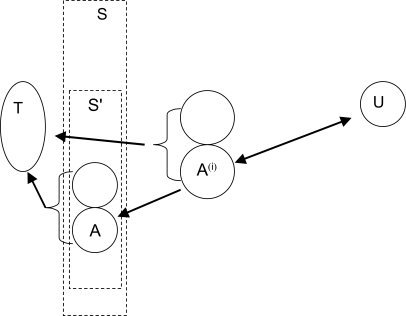
Procedure for finding ancestral tetraploid. *T* = genome made up of duplicated markers, *U* = reference genome. **S** = set of solutions to the genome halving problem. **S**′= subset closest to *U*, *A*^(^*^i^*^)^ = genome on trajectory from *A* ∈ **S**′ to *U*.

Let **S** be the set of solutions of the genome halving algorithm for *T*. As an initial step to our heuristic, schematized in Figure 3, we confine our search to **S**.

For each solution *A* ∈ **S**, we calculate the rearrangement distance *d* (*U, A*) between the reference genome *U* and *A*. This is feasible even for large **S** because of the rapidity of the rearrangement calculation. We then define

(1) $$S^{\prime }=\{A\in S|d\;(U,A)=\underset{X\in S}{\text{min}}d(U,X)\}.$$

By definition, there is no minimizing genome in **S**\**S**′.

To look for a better genome outside of **S**, for each *A* ∈ **S**′, we assume that any such genome will be found on a path between some element of **S**′ and *U*. We calculate the *d* (*U, A*) genomes, other than *A*, on a parsimonious trajectory *A, A*^(1)^, *A*^(2)^,···, *U* from *A* to *U*. Note that *d* (*U, A*^(^*^i^*^)^) = *d* (*U, A*) − *i*. Then we search for an *A*^(^*^i^*^)^ such that

(2) $$\begin{array}{l} d(U,A^{\left(i\right)})+d(A^{\left(i\right)}⊕A^{\left(i\right)},T)\\ <d(U,A)+d(A⊕A,T). \end{array}$$

(Note that it is not necessary to try *A*^(1)^ though it is closer by one step to *U* than *A* is, because *A*^(1)^ ⊕*A*^(1)^ is also farther from *T* by at least one step, since it is not in **S**.) Our final solution set **S**′′ is the set of *A*^(^*^i^*^)^, over all genomes *A* ∈ **S**′, and all trajectories between *A* and *U*, that satisfy inequality (2) and that minimize the left hand side of (2).

If **S**″ is empty, then **S**′ is the final set of minimizing genomes.

### Complexity

Since both genome halving and genome rearrangement are essentially linear in *n*, the execution time of our search is *O* (*n*|**S**| + φ*n*^2^| **S**′|), the second term measuring the number of steps between genomes in **S**′ and *U* and the time to calculate the distance to *U* at each step, and the number φ of different paths sampled per element in **S**′. In our example, biological reality motivates constraining the search so that all chromosome fissions are carried out first, as far as compatible with the optimality of the path. This is because the loss of chromosomes is likely to occur around the time of diploidization, so the path back from *A* towards the ancestor should attempt to restore the number of chromosomes to what it is in sorghum or rice as soon as possible, i.e. for some *A*^(^*^i^*^)^, where *i* is as small as possible.

## Results

The genome halving algorithm usually involves some arbitrary choices in constructing the optimal ancestral tetraploid. In the case of the maize genome, this leads to more than 5,000,000 different execution paths for the algorithm. Not all of these lead to the different results, but distinct solutions in **S** surely number in the hundreds of thousands, if not millions; a sample of 15,000 paths resulted in over 13,000 different solutions. The original data set not being very large (34 blocks in two genomes, 68 in maize), this exemplifies the extreme lack of uniqueness in the results of genome halving.

**Figure 4:**
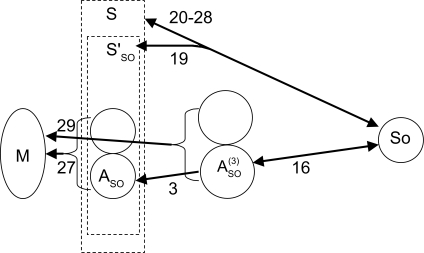

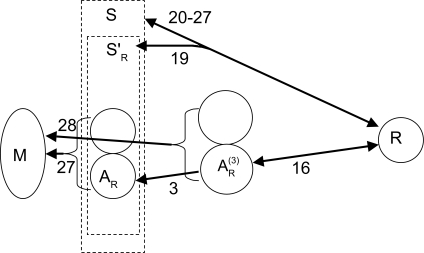
Results of search for ancestral tetraploid. *M, R, SO* = maize, rice, sorghum genomes. **S** = set of solutions to the genome halving problem. **S**′_R_, **S**′_SO_ = subsets closest to *R*, *SO*, *A_R_*^(^*^i^*^)^= genome on trajectory from *A_R_* ∈ **S**′ to *R. A_SO_*^(^*^i^*^)^ = genome on trajectory from *A_SO_* ∈ S′_SO_ to *SO*.

**Figure 5:**
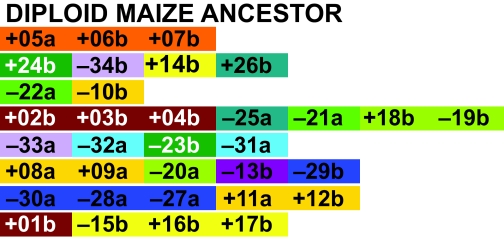

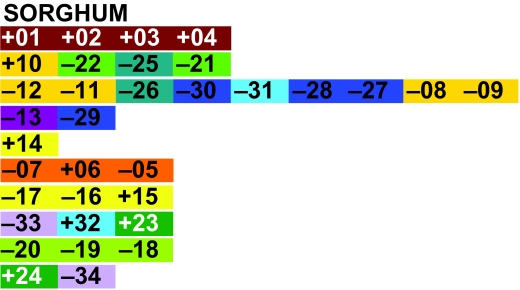
Order of syntenic blocks in the reconstructed diploid maize ancestor, compared to sorghum, with the same rice chromosomal colour coding as in Figure 2.

When we bring the reference genomes to bear, we first note that over all *X* ∈ ***S***, the distance *d* (*X, So*) ranges from 19 (for the solutions in **S**′_SO_) to 28, while *d* (*X, R*) ranges from 19 (for the solutions in **S**′ _R_) to 27. The sets **S**′ _SO_ and **S**′_R_, however, contain only 8 and 24 solutions, respectively. Thus there is a massive reduction of non-uniqueness induced by appealing to a reference. Then, in venturing outside of **S** on the paths from pre-tetraploid versions of elements of **S**′ towards the reference, either rice or sorghum, we find even fewer genomes *X* with a minimum sum of distance to the reference (*X* as a diploid) plus distance to maize (*X* ⊕ *X* as a tetraploid). For example, the genome *A_SO_*^(3)^ in Figure 4 and depicted in Figure 5 satisfies

(3) $$\begin{array}{l} d(So,A_{SO}^{\left(3\right)})+d(A_{SO}^{\left(3\right)}⊕A_{SO}^{\left(3\right)},M)=16+29\\ <d(So,A)+d(A⊕A,M)=19+27, \end{array}$$

inequality (3) for all *A* ∈ **S**′. There are only two other solutions with value 45 for the objective function, one a step closer (an *A*^(4)^) and one a step further (an *A*^(2)^), from the sorghum genome. In the case of a rice reference, there is actually a unique solution, with *d*(*R, X*) + *d* (*X* ⊕ *X*, *M*) = 44.

Thus we have almost completely eliminated the non-uniqueness of the solutions to the genome-halving problem, though of course the number of solutions found will still depend on the data set. It is also possible that a better solution is to be found off the paths we have explored, although this is unlikely for the relatively small example represented by these cereal genomes.

## Conclusions

We have been working with a small data set, and the differences between the optimal solution and suboptimal solutions are small, as in inequality (3). As more data become available on maize and especially sorghum, our reconstructions should be better and the role of the reference genome in zeroing in on a unique solution for genome halving will be clarified. This should also allow for statistical validation.

Our analysis used sorghum and rice as reference genomes in two separate analyses. And it is gratifying that using sorghum alone as reference produced an ancestral maize genome closer, not only to sorghum, but also to rice, than any candidate ancestor based on genome halving with no reference. Nevertheless, it would be interesting to formally combine gene order information from both rice and sorghum simultaneously in reconstructing the maize ancestor. Along the lines of our current analysis, first finding **S**, then **S**′, and finally an optimal *A*^(^*^i^*^)^, we could define **S**′ as the subset of **S** whose elements *A* each induce a minimal solution of the median problem (Sankoff and Blanchette, 1997; Siepel, 2001), i.e. for which there is a genome *X*, such that *d* (*A, X*) + *d* (*U*_1_, *X*) + *d* (*U*_2_, *X*) is minimal compared to all *A* ∈ ***S***. Then the search for an optimal *A*^(^*^i^*^)^ could proceed on the paths from all *A* ∈ **S**′ to *X*.

A more difficult theoretical problem would be to replace our sequential procedure by a single algorithm searching for the *A* which minimizes *d* (*U, A*) + *d* (*A* ⊕ *A,T*). It is not clear whether this is a hard problem, given that genome halving and genome rearrangement are both solvable in close to linear time. But there is no obvious way of modifying the halving algorithm so that it could take account of a reference genome while retaining optimality. Some of the searches we have performed here might be incorporated directly into the halving algorithm to transform it into a heuristic method, and this might work even for the direct minimization of *d* (*U*_1_, *A*) + *d* (*U*_2_, *A*) + *d* (*A* ⊕ *A,T*).
